# Multi systemic compromise due to *Bartonella henselae* in a child

**DOI:** 10.1590/S1678-9946202567047

**Published:** 2025-07-18

**Authors:** Thiago Jessé Kucarz, Cintia Avila Souza, Simone Aiko Hatanaka, Elisa Nunes Secamilli, Marina Rovani Drummond, Marcos Tadeu Nolasco da Silva, Rafael Fantelli Stelini, Maria Leticia Cintra, Paulo Eduardo Neves Ferreira Velho

**Affiliations:** 1 Universidade Estadual de Campinas, Faculdade de Ciências Médicas, Departamento de Clínica Médica, Disciplina de Dermatologia, Campinas, São Paulo, Brazil; 2 Universidade Estadual de Campinas, Faculdade de Ciências Médicas, Laboratório de Pesquisa Aplicada em Dermatologia e Infecção por Bartonella, Campinas, São Paulo, Brazil; 3 Universidade Estadual de Campinas, Faculdade de Ciências Médicas, Departamento de Pediatria, Campinas, São Paulo, Brazil; 4 Universidade Estadual de Campinas, Faculdade de Ciências Médicas, Departamento de Patologia, Divisão de Anatomia Patológica, Campinas, São Paulo, Brazil

**Keywords:** Bartonella, Vasculitis, Epididymitis, Arthritis, Bartonella infections

## Abstract

Currently, at least 22 species of *Bartonella* are known to cause diseases in humans, with *Bartonella henselae* being the main one. Among the clinical manifestations associated with bartonellosis, cutaneous vasculitis is rare, but it can be severe. We report the case of a 9-year-old child who presented with cervical lymphadenopathy, arthritis, epididymitis, and cutaneous vasculitis as clinical manifestations of systemic bartonellosis, with positive detection of *B. henselae* in blood and skin fragment using species-specific conventional polymerase chain reaction (PCR) techniques. Vasculitis caused by *Bartonella* spp. occurs due to the endothelial tropism of the bacteria and can mimic systemic vasculitis with positive anti-neutrophil cytoplasmic antibodies (ANCA). Furthermore, we found just one previous report about epididymitis related to *B. henselae* infection, and arthritis is also considered an unusual manifestation of the infection. This case emphasizes the need to consider bartonellosis among differential diagnoses when faced with presentations of purpura, cutaneous vasculitis, arthritis, or epididymitis.

## INTRODUCTION

*Bartonella* species are intracellular bacilli responsible for prolonged and recurrent infections^1^. Currently, at least 22 pathogenic species are known for infecting humans, with *Bartonella henselae* being the most relevant agent^2,3^. The main vectors of these bacteria are hematophagous arthropods, such as fleas and ticks. Felines and canines serve as reservoirs for this species; having contact with these animals is considered a risk factor for infection, which can manifest asymptomatically or even result in death^4–6^. Clinical manifestations associated with bartonellosis include fever, anemia, hepatitis, serositis, endocarditis, lymphadenopathy, uveitis, retinitis, neuritis etc. Dermatological manifestations are diverse, including purpura, vasculitis, maculopapular rash, urticaria, erythema nodosum, erythema marginatum, erythema multiforme, granuloma annulare, as well as granulomatous and angioproliferative reactions^4^. The objective of this study is to report the case of a child who presented cutaneous vasculitis, arthritis, and epididymitis associated with *B. henselae* infection, which was cured after appropriate antibiotic therapy.

## CASE REPORT

**Figure 1:**
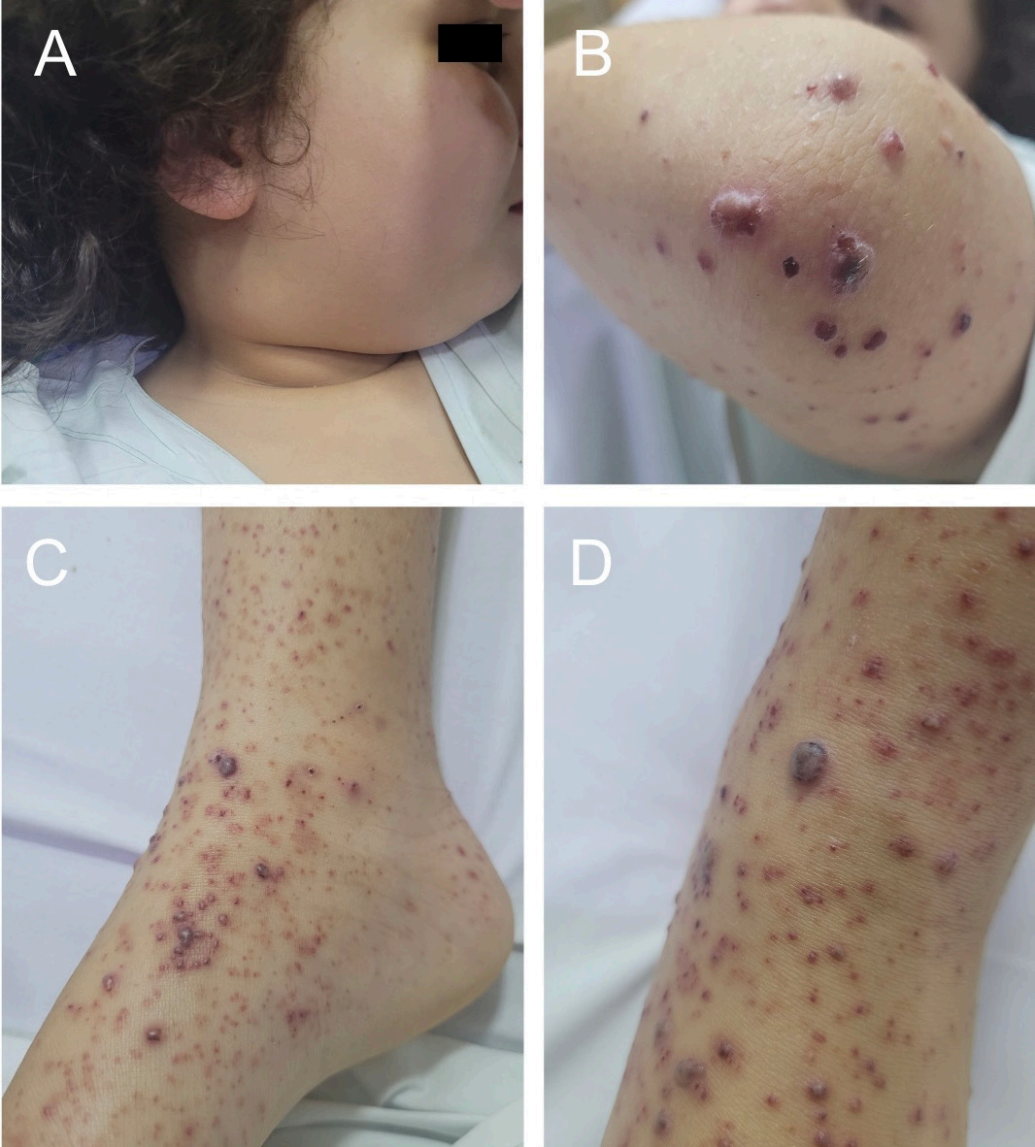
Initial clinical presentation: (A) Right cervical lymphadenopathy; (B, C, and D) palpable and nonpalpable purpura on the left elbow, right lower limb, and left lower limb, respectively.

**Figure 2:**
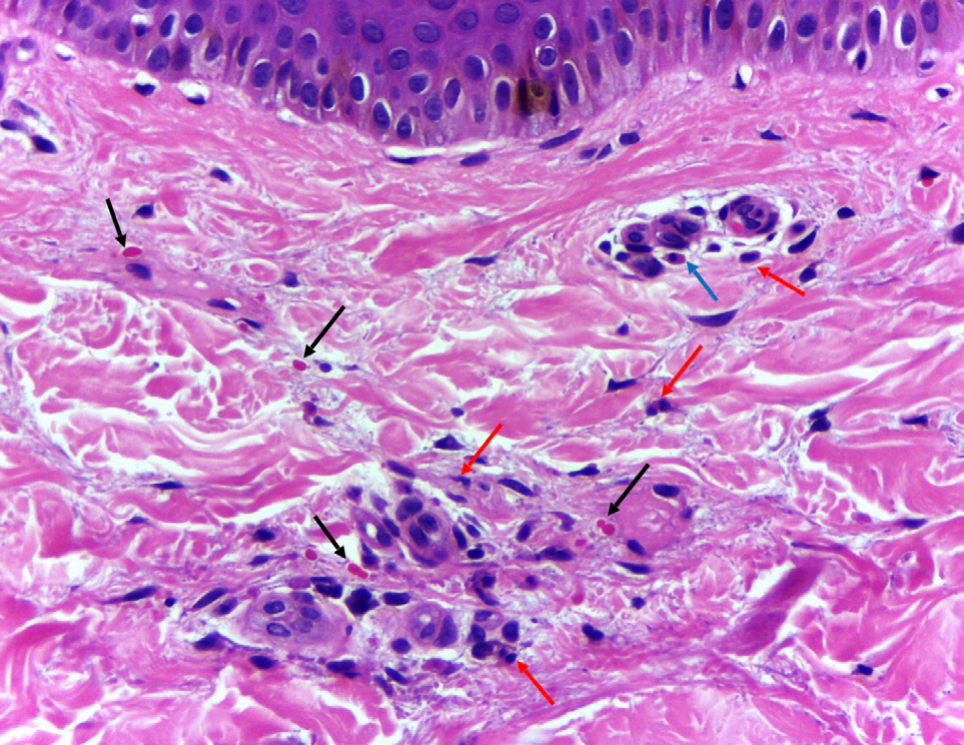
Histopathological examination of skin fragment showing extravasated red blood cells (black arrows), sparse neutrophils (red arrows) and eosinophils (blue arrow) in the perivascular region.

**Figure 3:**
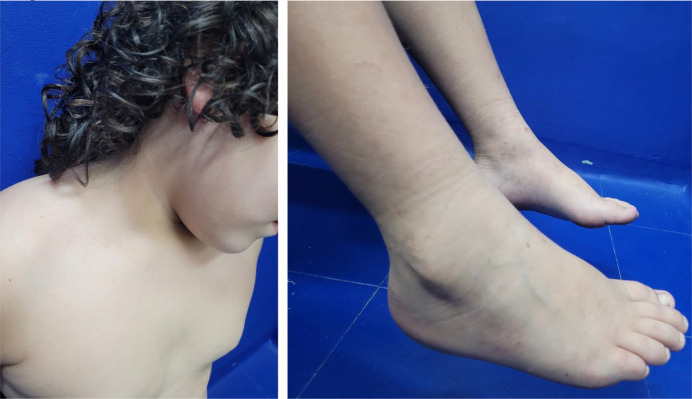
Clinical presentation after treatment, with improvement of cervical lymphadenopathy and purpura in the lower limbs.

The patient, a 9-year-old boy, was admitted with complaints of pain in the lower limbs, swelling in the right cervical region, and lesions that progressed from the lower to the upper limbs, with onset 10 days prior. He did not report recent weight loss. The patient and his family were previously healthy and lived in a rural area of Brazil, having contact with cats, dogs, pigs, chickens, and wild animals. He denied any history of scratches or bites from animals. On physical examination, a palpable cervical mass was observed, non-adherent, fibroelastic, painful, approximately 5 cm in diameter on the right side, with palpable, mobile lymph nodes about 1 cm in diameter on the left side. In the upper and lower limbs, palpable and non-palpable purpura were noted (Figure 1). Furthermore, the patient presented edema, local warmth, and pain on mobilization of the right ankle and knee. The left testicle showed increased volume and tenderness on palpation. During hospitalization, the patient experienced a low-grade fever episode. The ultrasound of the scrotum revealed a retractile testicle on the right, with no visualization of the right epididymis and evidence of left epididymitis. The computed tomography of the neck demonstrated bilateral cervical lymphadenopathy, especially at levels II to IV, with several cervical lymph nodes showing necrotic areas and moderate retropharyngeal edema. Rapid testing for Sars-CoV-2, two blood cultures, and a urine culture were negative. Serologies for toxoplasmosis, cytomegalovirus, and mononucleosis were non-reactive (IgG and IgM). Leukocytosis of 24,160 cells/μL with a left shift and c-reactive protein (CRP) level of 215 mg/L were noted, with no other significant laboratory alterations. The anatomopathological examination of the skin fragment revealed extravasated erythrocytes and the presence of neutrophils and eosinophils in the perivascular region (Figure 2). Direct immunofluorescence showed negative results for IgA, IgG, C1q, and C3, with IgM demonstrating adsorption in sparse apoptotic keratinocytes. The detection of *B. henselae* in blood and skin fragments by conventional PCR, specific for the *gltA* gene, identified the target DNA in both samples, confirming the diagnosis of cutaneous vasculitis associated with *B. henselae* infection. Treatment during hospitalization included doxycycline 5 mg/kg/day, clindamycin 40 mg/kg/day, and amikacin 15 mg/kg/day for 8 days. The patient was discharged with doxycycline 10 mg/kg/day for three months. He progressed with complete improvement of cervical lymphadenopathy, purpuric lesions, arthritis, and epididymitis (Figure 3). Currently, he has regular outpatient follow-up and has not experienced recurrences of the condition 20 months after completing treatment.

The case was conducted in accordance with the Ethics Code of the World Medical Association (Declaration of Helsinki). The minor's legal guardian signed the informed consent form and the child signed the informed assent form.

## DISCUSSION

In *B. henselae* infection, purpura, although rare, can be severe and associated with fever and lymphadenopathy, which were present in our patient^7^. Vasculitis caused by *Bartonella* spp. occurs due to the endovascular tropism of the bacteria and can simulate systemic vasculitis with positive antineutrophil cytoplasmic antibodies (ANCA)^3,7^. Although this test was requested for our patient, it could not be performed at the hospital.

*Bartonella* spp. can cause asymptomatic cyclic bacteremia in humans and animals^4,8^. Approximately 31% of endocarditis cases are culture-negative, and of these, up to 30% are caused by *Bartonella* spp.^9,10^. In renal involvement associated with *B. henselae* infections, the most common pattern is that of diffuse proliferative post-infectious glomerulonephritis secondary to immune complex deposition, typically linked to native-valve endocarditis^11^. The investigation for renal involvement was conducted in our patient and no alterations were found.

The laboratory diagnosis of infections caused by *Bartonella* spp. is challenging. Serology with indirect immunofluorescence may indicate past infection and can present cross-reactivity with other pathogens, leading to false-positive results. Additionally, the antigens in commercial kits are restricted to a few species, resulting in false-negative outcomes as well^12,13^. However, indirect immunofluorescence is recommended in settings in which molecular tests are not routinely available^14^. Histology can diagnose classic bartonellosis, such as bacillary angiomatosis or cat scratch disease^4^. Culture requires special conditions, and even under these conditions, primary isolation is rare and it can take six to eight weeks to obtain the isolate^15^. Detection of *Bartonella* spp. using the PCR technique (from blood or tissue) provides a faster result and enables species identification, as in the reported case, in which the result was positive in both the blood and skin fragment, identifying *B. henselae* ^16^. Multiple techniques should be used in combination to avoid false-negative results^9^. These difficulties are another factor that contribute to misdiagnosing the condition^4^.

There is no treatment that guarantees eradication of the agent from the host. Systemic disease, similar to endocarditis caused by these agents, could be treated with an aminoglycoside for two weeks and doxycycline for six weeks to six months, although there are no studies supporting this or other therapeutic regimens^3^. Other treatment options include macrolides, sulfas, and rifampicin^17^. Our patient received clindamycin and amikacin for eight days and doxycycline for three months, showing no recurrence of the condition after medication cessation in 20 months of follow-up.

Vasculitis caused by *Bartonella* spp. can mimic primary vasculitis, a possibility that should be considered in the differential diagnosis of purpura or vasculitis.^7^ Furthermore, the authors found just one previous report about epididymitis related to *B. henselae* infection, and arthritis is also considered an unusual manifestation of the infection^18,19^.

## CONCLUSION

The reported case involves a child with an epidemiological background for bartonellosis, cervical lymphadenopathy, low fever, arthritis, epididymitis, and cutaneous vasculitis. The disease was considered as a differential diagnosis, appropriate diagnostic techniques were utilized, and the correct therapy was instituted, resulting in the resolution of the condition. Definitive diagnosis of bartonellosis remains challenging due to technical and analytical limitations, as well as the condition often being overlooked in clinical settings.
